# GDF15 contributes to radiation-induced senescence through the ROS-mediated p16 pathway in human endothelial cells

**DOI:** 10.18632/oncotarget.7457

**Published:** 2016-02-17

**Authors:** Hyejin Park, Chun-Ho Kim, Jae-Hoon Jeong, Myungjin Park, Kwang Seok Kim

**Affiliations:** ^1^ Divisions of Radiation Effects, Korea Institute of Radiological and Medical Sciences, Seoul, Republic of Korea; ^2^ Research Center for Radiotherapy, Korea Institute of Radiological and Medical Sciences, Seoul, Republic of Korea; ^3^ Divisions of Radiation Cancer Science, Korea Institute of Radiological and Medical Sciences, Seoul, Republic of Korea

**Keywords:** cellular senescence, ionizing radiation, p53, p16, endothelilal cells, oxidative stress, Gerotarget

## Abstract

Growth differentiation factor 15 (GDF15) is an emerging biomarker of cardiovascular risk and disease. Microarray analyses revealed that GDF15 levels were increased during cellular senescence induced by ionizing radiation (IR) in human aortic endothelial cells (HAECs). However, the role of GDF15 in HAEC cellular senescence remains unclear. This study demonstrated that downregulation of GDF15 in HAECs partially prevented cellular senescence triggered by IR, which was confirmed by recovery of cell proliferation and reverse senescence-associated β-galactosidase (SA-β-gal) staining. Conversely, upregulation of GDF15-induced cellular senescence in HAECs, confirmed by G0/G1 cell cycle arrest, decreased during cell proliferation and increased SA-β-gal staining. GDF15-induced cellular senescence was observed in p16-knockdown cells but not in p53-knockdown cells. GDF15 expression in endothelial cells also generated reactive oxygen species (ROS), which led to activation of extracellular signal-regulated kinases (ERKs) and induction of senescence by oxidative stress. These results suggested that GDF15 might play an important role in cellular senescence through a ROS-mediated p16 pathway and contribute to the pathogenesis of atherosclerosis via pro-senescent activity.

## INTRODUCTION

Cellular senescence is a stress-response phenomenon in which cells reach a state of irreversible arrest that involves cellular changes. Irreversible growth arrest is induced by accumulation of DNA damage, epigenetic de-repression of the critical tumor suppressor genes p16^INK4a^ and p19^ARF^, or by telomere shortening [1]. These mechanisms induce senescence by p53 and p16/Rb, which commonly function as primary effectors of cellular senescence in normal somatic cells [2-3].

Growth differentiation factor 15 (GDF15) is currently being evaluated as a biomarker of cardiovascular stress and diseases that are linked to the incidence, progression, and prognosis of heart failure [4]. GDF15 has also been shown to be a strong and independent predictor of mortality and disease progression in patients with established disease [5-6].

GDF15 is a member of the transforming growth factor β superfamily. The expression of GDF15 occurs in virtually all tissues, which suggests its importance in general and basic cellular functions. The expression of GDF15 is also upregulated in many different pathological or metabolic conditions, including inflammation [7], mitochondrial disease [8], impaired fasting glucose [9], and invasion and metastasis of certain cancers [10]. However, the exact biological functions of GDF15 are still poorly understood, and its function often exhibits differing and sometimes opposing roles under various circumstances [6, 11]. Consequently, GDF15 exhibits a complex pattern of beneficial and harmful functions.

Our study investigated whether or not increased GDF15 can cause certain cellular damage or induce cellular senescence by response to oxidative stresses such as ionizing radiation (IR). The present study demonstrated that the GDF15 expression levels were increased in both replicative senescence and premature senescence with IR treatment of human aortic endothelial cells (HAECs). To test the role of GDF15 in endothelial senescence, we evaluated if the downregulation of GDF15 reversed cellular senescence in HAECs and if GDF15-induced senescence was associated with two critical senescence effectors, the p53/p21 or p16/Rb pathways.

## RESULTS

### GDF15 expression increases in IR-induced senescence of endothelial cells

**Figure 1:**
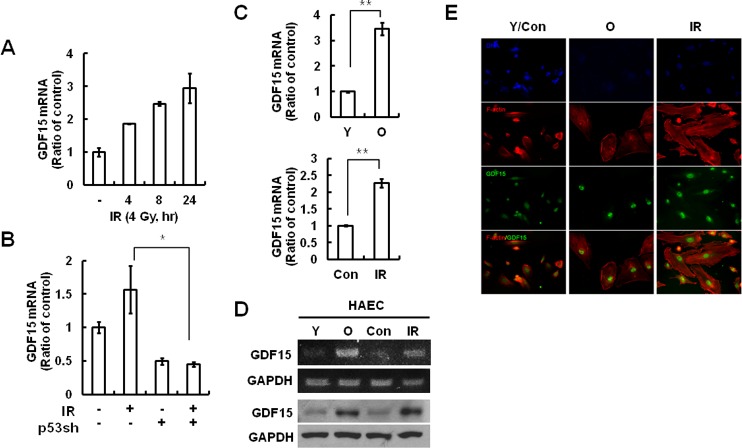
GDF15 expression levels in IR-induced endothelial cell senescence **A.** Cells were treated with IR for 0, 4, 8, or 24 h, and the expression of GDF15 mRNA levels was measured by real-time-PCR. **B.** Cells were transduced with a p53sh retrovirus and then treated with IR for 24 h. RNA was purified from the cells and the expression levels of GDF15 were measured by real-time-PCR. * = *p* < 0.05 *versus* IR only treatment. **C.** RNA was extracted from young or old cells and the IR-untreated or the 48 h IR-treated group, and the GDF15 expression levels were confirmed by real time-PCR. Values are means ± SD of three experiments. ** = *p* < 0.01 *versus* young cells or the control group. **D.** Target sequences for GDF15 were amplified by semi-quantitative PCR and the amplified DNAs were analyzed by agarose gel electrophoresis. The GDF15 protein levels were confirmed by western blotting. Representative data from three independent experiments are shown. **E.** The cells were stained with rhodamine-conjugated phalloidin (*red*) for actin, FITC-conjugated antibody for GDF15 (*green*) and DAPI (*blue*) for nuclei, and were observed using a fluorescence microscope (200×). Y = young cells; O = old cells; Con = control group; IR = ionizing radiation-treated group.

Microarray analyses revealed that GDF15 expression increased following exposure to IR (data not shown). We confirmed GDF15 expression was increased in both dose- and time-dependent manners (Figure 1A). IR primarily triggers DNA damage responses in human cells, of which p53 remains a central player. To determine whether p53 increases GDF15 expression in response to DNA damage following IR exposure, GDF15 was measured post-irradiation in p53-depleted cells. IR-induced GDF15 expression resulted from p53-dependent transcriptional regulation (Figure 1B). Expression of GDF15 was increased (Figure 1C). To investigate whether GDF15 was associated with IR-induced cellular senescence in endothelial cells, we examined GDF15 expression levels in young and old cells by semi-quantitative PCR and real-time PCR. GDF15 mRNA levels were upregulated in older endothelial cells, as well as after radiation exposure (Figure 1C and 1D). The increased expression of GDF15 during both aging and irradiation was confirmed using fluorescence microscopy (Figure 1E).

### Reversal of IR-induced senescence by GDF15 knockdown in HAECs

**Figure 2:**
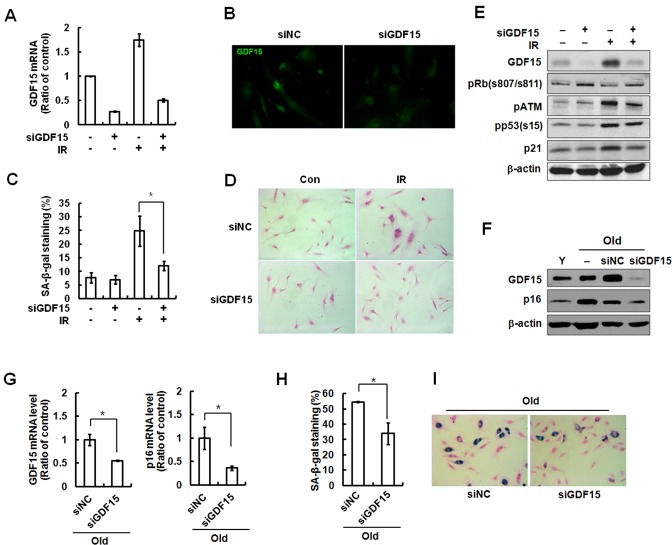
Reversal of cellular senescence following knockdown of GDF15 in HAECs **A.** Cells were transfected with GDF15 siRNA and then treated with IR for 4 days. The GDF15 expression levels were confirmed by real-time-PCR. **B.** The cells were stained with FITC-GDF15 antibody and observed using a fluorescence microscope (200x). **C.** Cells were stained for SA-β-gal activity and the percentage of blue cells per every 400 cells was calculated. Values are means ± SD of three independent experiments. * = *p* < 0.05 *versus* IR only treatment. **D.** SA-β-gal staining of cells was evaluated using a light microscope (100×). **E.** Phosphorylation of Rb at serine 807/serine 811, p53 at serine 15, ATM at serine 1981, p21, and GDF15 were analyzed by western blotting. β-actin was used as a protein loading control. Representative data from three independent experiments are shown. **F.** GDF15 was downregulated by GDF15 siRNA in old HAECs. GDF15 and p16 were analyzed by western blotting. **G.** The expression levels of GDF15 and p16 were measured by real-time-PCR. * = *p* < 0.05 *versus* the negative control (NC) siRNA group. **H.**, **I.**. SA-β-gal staining was performed in the old cells and SA-β-gal activity (%) was shown. Values are means ± SD of three independent experiments. * = *p* < 0.05 *versus* the NC group.

Several phenotypes were induced by IR exposure in vascular cells, such as a decrease in mitogen-induced proliferation, expression of SA-β-gal, and a characteristically enlarged and flattened morphology. To investigate the role of GDF15 in cellular senescence, we tested the effect of GDF15 downregulation using GDF15 siRNA during premature senescence triggered by IR treatment. Following GDF15 siRNA, GDF15 expression was silenced in approximately 75% of the young HAECs (Figure 2A), which was apparent by decreased fluorescence intensity of GDF15 (Figure 1B). GDF15-downregulated cells were protected from IR-induced senescence, as evidenced by a reduction in SA-β-gal staining (Figure 2C and 2D). Expression of phospho-ATM, p53, and p21 proteins following IR treatment was partially reduced by the transfection of GDF15 siRNA, but the changes were not significant (Figure 2E). Major effects on IR-induced DNA damage response resulted from the p53 pathway *via* activation of ATM, but p53 was not directly regulated by GDF15 depletion. We also investigated the effect of GDF15 downregulation in older cells. Repression of GDF15 levels decreased the expression of p16 protein, which was high in older cells (Figure 2F). Additionally, p16 mRNA levels were decreased by GDF15 downregulation (Figure 2G). As well, a decrease in SA-β-gal staining was shown after GDF15 depletion (Figure 2H and 2I). These results suggested that knockdown of GDF15 in older cells and in IR-treated cells partially reversed senescence phenotypes, but did not overcome DNA damage responses *via* the p53/p21 pathway.

### Effects of GDF15 upregulation on cellular senescence of HAECs

**Figure 3:**
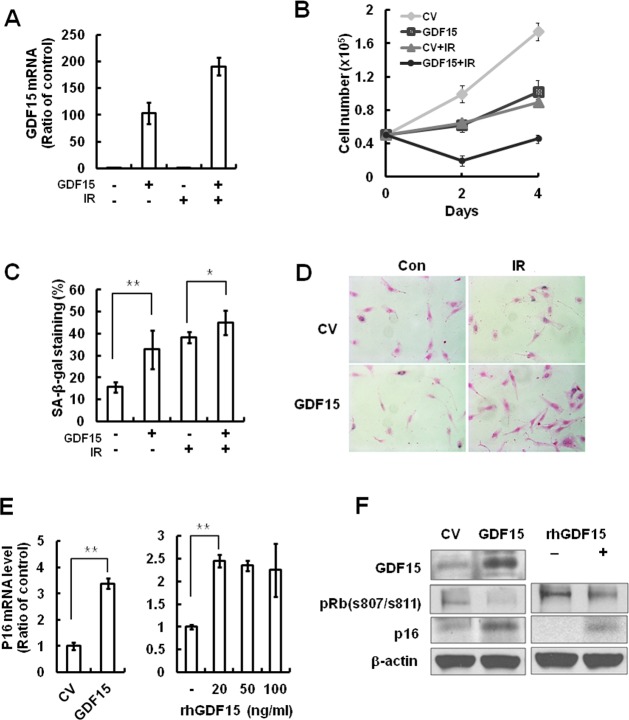
The effects of GDF15 overexpression on cellular senescence in HAECs **A.** Cells were transduced with GDF15 lentivirus and then treated with IR for 4 days. The GDF15 expression levels were confirmed by real-time-PCR. **B.** Cell proliferation was measured by counting cell numbers for 4 days. Values are means ± SD of three experiments. **C.**, **D.** Cells were stained for SA-β-gal activity and the percentage of blue cells per every 400 cells was calculated. Values are means ± SD of three independent experiments. ** = *p* < 0.01 *versus* the control group and * = *p* < 0.05 *versus* the IR only treated group. **E.** The p16 mRNA expression levels were measured by GDF15 overexpression or by treatment with 0, 20, 50, or 100 ng/ml rhGDF15. ** = *p* < 0.01 *versus* the control group. **F.** GDF15, phospho-Rb (s807/s811), and p16 were confirmed by western blotting in GDF15-transduced cells or rhGDF15-treated cells. β-actin was used as a protein loading control. Representative data from three independent experiments are shown.

Because GDF15 expression levels were increased in senescent cells and following IR treatment, we tested whether GDF15 overexpression had an impact on cellular senescence in HAECs. HAECs were transduced with a GDF15 lentivirus and senescence markers in cells overexpressing GDF15 were examined. Upregulation of GDF15 caused a decrease in cell proliferation (Figure 3B) and an increase in SA-β-gal staining compared with the control lentivirus-transduced cells (Figure 3C and 3D). Increased expression of GDF15 induced p16 expression (Figure 3D) and treatment with GDF15 recombinant protein increased p16 mRNA by approximately 2.5 fold (Figure 3E). Both endogenous and exogenous GDF15 protein increased p16 protein and decreased the phosphorylation of Rb, which causes its detachment from E2F transcription factor (Figure 3F). Taken together, these results suggested that GDF15 might play an important role in cellular senescence in HAECs *via* the expression of p16.

### Induction of cellular senescence by GDF15 *via* a p16 signaling pathway

**Figure 4:**
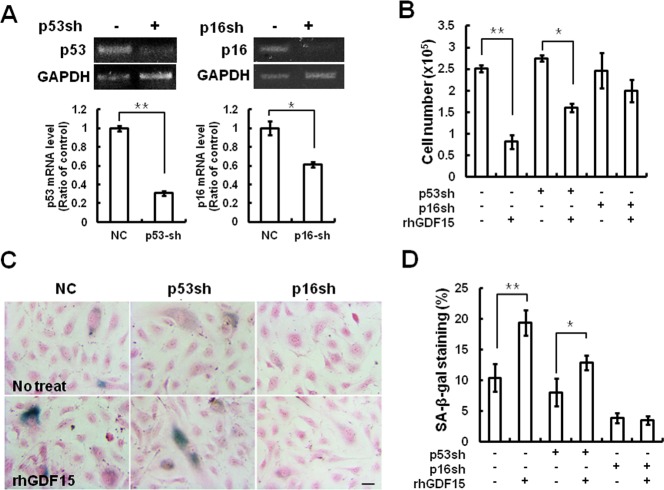
Cellular senescence induced by GDF15 *via* the p16 signaling pathway **A.** HAECs were transduced with a p16 or p53 shRNA retrovirus. RNA was extracted from the cells and knockdown of p16 or p53 mRNA levels was confirmed by RT-PCR and real-time-PCR. * = *p* < 0.05 and ** = *p* < 0.01 *versus* the NC group. **B.** The p16 or p53 shRNA cells were treated with 100 ng/ml GDF15 protein for 6 days and cell number counting was performed to determine cell proliferation. **C.**, **D.** SA-β-gal-positive cells were measured. Values are means ± SD of three independent experiments. ** = *p* < 0.01 *versus* the NC group and * = *p* < 0.05 *versus* the p53sh group.

Because p16^INK4a^ (CDKN2A) can be induced by stress, but not by DNA damage or inflammatory secretion, and can stimulate the Rb-regulated growth arrest [12], it is reasonable to expect that GDF15 activates specific signaling pathways to engage p53/p21 or/and p16/Rb proteins. To determine which pathway was involved in cellular senescence induced by GDF15, we performed a knockdown of p16 or p53 using shRNA retroviruses in HAECs and measured the effects of GDF15 on cellular senescence. The expression levels of p53 or p16 in HAECs with each shRNA retrovirus were confirmed by semi-quantitative PCR and real-time PCR (Figure 4A). The p53 knockdown cells exhibited decreased cellular proliferation by the overexpression of GDF15, which was similar to the control cells. On the contrary, the overexpression of GDF15 had no significant effects on cell proliferation in the p16 knockdown cells (Figure 4B). The measurement of SA-β-gal activity indicated that p16 knockdown inhibited GDF15-induced cellular senescence, but p53 knockdown did not (Figure 4C, 4D). Therefore, these results suggested that cellular senescence induced by GDF15 might be increased through a p16/Rb-dependent pathway.

### Generation of ROS by GDF15 induction

**Figure 5:**
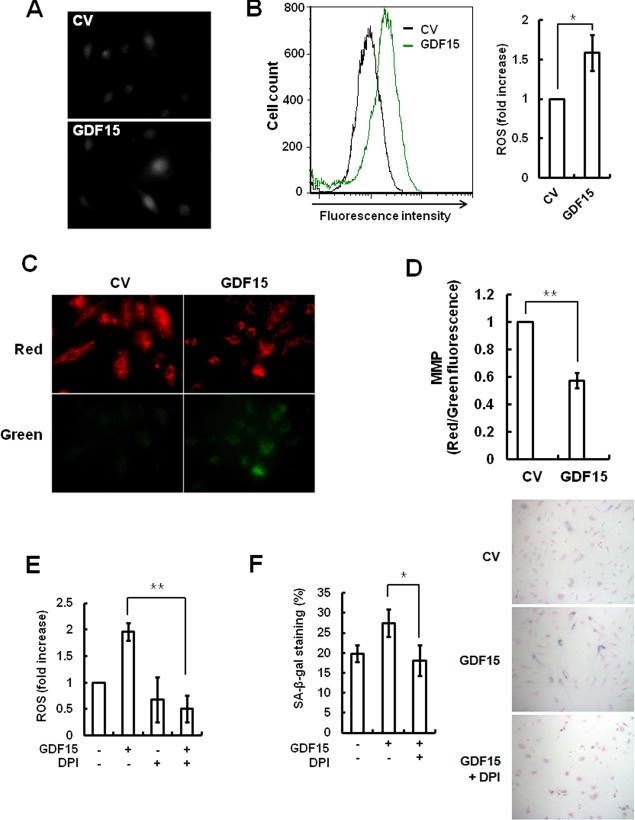
ROS generation following induction of GDF15 Cells were transduced with GDF15 lentivirus and ROS generation was analyzed with DCFDA by fluorescence microscopy **A.** and by flow cytometry **B.**. * = *p* < 0.05 *versus versus* the control group. **C.** Mitochondrial membrane potential of cells was analyzed by JC-1 fluorescence. **D.** The ratio of red/green fluorescence with JC-1 was measured by FACS analysis. ** = *p* < 0.01 *versus* the control group. **E.** GDF15-transduced cells were treated with 10 μM DPI for 24 h and ROS generation was analyzed by flow cytometry using DCFDA. ** = *p* < 0.01 *versus* the GDF15 group. **F.** SA-β-gal-activity was measured in the cells. Values are means ± SD of three independent experiments. * = *p* < 0.01 *versus* the GDF15 group.

Because GDF15 can induce endothelial senescence *via* the p16 pathway, which is operated by oxidative stress, we tested whether GDF15 was associated with ROS-induced senescence and could generate ROS. The results of the fluorescence microscopy measurements involving DCFDA indicated that ROS generation was increased in GDF15-tranduced cells compared to control virus-transduced cells (Figure 5A). We confirmed and quantified the accumulation of ROS detected by immunofluorescence using flow cytometry with cells that were transduced with GDF15 virus (Figure 5B). In order to evaluate Mitochondrial Membrane Potential (MMP), GDF15-tranduced cells were stained with the cationic dye JC-1, which revealed red aggregates for normal mitochondrial membranes and green monomers upon membrane depolarization. The appearance of green monomers was increased in mitochondria in the GDF15-transduced cells, which indicated increased mitochondrial dysfunction (Figure 5C), and reductions in MMP caused by GDF15 were more than double compared to normal conditions (Figure 5D). Diphenyleneiodonium (DPI) is an NADPH oxidase inhibitor that can inhibit mitochondrial ROS generation. Treatment with DPI inhibited GDF15-induced generation of ROS (Figure 5E). As well, SA-β-gal staining revealed that GDF15-induced ROS generation was correlated to cellular senescence (Figure 5F). Taken together, the results indicated that mitochondrial ROS generation by GDF15 was involved in cellular senescence in HAECs.

### Involvement of ERK activation in GDF15-induced senescence through the ROS-mediated p16 pathway

**Figure 6:**
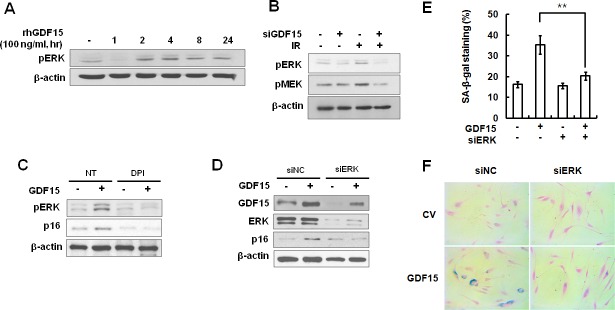
Activation of ERK by GDF15 through the generation of ROS **A.** HAECs were treated with 100 ng/ml rhGDF15 for 0, 1, 2, 4, 8, or 24 h. Activation of ERK was analyzed by western blotting. **B.** Cells were transfected with GDF15 siRNA and treated with IR for 24 h. Phosphorylation of ERK and MEK was analyzed by western blotting. **C.** Cells were transduced with GDF15 lentivirus and treated with 10 μM DPI for 24 h. Phospho-ERK and p16 protein were analyzed by western blotting. Representative data from three independent experiments are shown. **D.** GDF15-transduced cells were transfected with ERK siRNA for 36 h and GDF15, ERK, and p16 protein were analyzed by western blotting. **E.**, **F.**. SA-β-gal activity was measured in the cells. Values are means ± SD of three independent experiments. ** = *p* < 0.01 *versus* the GDF15 group.

Activation of mitogen-activated protein kinases (MAPKs), such as p38 and ERK, plays a role in inducing senescence in response to oxidative stress [13]. We examined the levels of activated MAPKs in GDF15-transduced HAECs and by treatment with rhGDF15 protein. We noted that ERK phosphorylation was increased following the rhGDF15 protein treatment (Figure 6A). In addition, IR-induced ERK activation was controlled by the downregulation of GDF15 (Figure 6B). In order to test whether mitochondrial ROS was implicated in GDF15-induced ERK activation, GDF15-transduced cells were treated with DPI. The results indicated that the apparent increase in ERK phosphorylation caused by GDF15 was inhibited by DPI treatment, which led to decreased p16 expression (Figure 6C). Further, ERK depletion with ERK siRNA inhibited GDF15-induced p16 expression (Figure 6D) and suppressed GDF15-associated increases in SA-β-gal activity (Figure 6E and 6F), which suggested that ERK activation was involved in GDF15-induced senescence. Taken together, these data suggested that GDF15 induced ROS-related ERK activation in cellular senescence.

## DISCUSSION

Because radiation is used increasingly for practical applications, the health risks of radiation exposure have to be considered. The damage to endothelial cells by IR includes changes in permeability, disruption of the cytoskeleton, angiogenic capacity impediments, and premature senescence [14]. Recent evidence suggests that endothelial cell senescence potentially contributes to vascular aging and aging-associated vascular diseases, particularly to the pathogenesis of human atherosclerosis [15-16]. Therefore, we have revealed that an IR-induced factor caused premature senescence in endothelial cells and was associated with the risk of cardiovascular disease.

The present study provided the first evidence of GDF15 involvement in cellular senescence of human primary endothelial cells through IR-induced damage. We demonstrated that GDF15 plays an important role in IR-induced senescence of endothelial cells by demonstrating that GDF15 expression was increased during replicative senescence or IR exposure (Figure 1). We also revealed that cellular senescence was reversed by the downregulation of GDF15 (Figure 2), and that GDF15 overexpression induced growth arrest (Figure 3) and cellular senescence, as confirmed by the upregulation of p16, changes in cell morphology, and increased SA-β-gal staining (Figures 3 and 4).

Accumulating evidence suggests that the p53 and p16/Rb tumor suppressor pathways are key regulators of the senescence response [17-18]. We also revealed that p16 was required for GDF15-induced senescence, which was indicated by our findings that GDF15 can regulate p16 expression (Figure 3) and that GDF15 increased SA-β-gal staining in p53-knockdown cells but not in p16-knockdown cells (Figure 4).

Endothelial cells are sensitive to stress-induced senescence and have both p16-dependent and p53-dependent pathways of senescence. Specifically, p16 is induced by oncogenes such as Ras [19]and Raf [20], as well as by oxidative stress signals [21], and is required for telomere-independent senescence [22]. Oncogene-induced senescence (OIS), which is typically mediated by Ras, activates the MAPK pathway. OIS is dependent on the involvement of the p53 and p16-Rb pathways, but p16 plays a more prominent role than p53, as some cells depend solely on p16 for OIS [18, 23]. Stress signals such as ROS stimulate the activation of p16 transcription and play important roles in initiation, as well as maintenance, of cellular senescence [24]. As well, ROS derived from mitochondria are involved in senescence, and mitochondrial ROS generation is associated with GDF15-induced senescence (Figure 6). Activation of p38 by the accumulation of ROS regulates p16 expression and cytoskeletal remodeling [25]. Our study revealed that GDF15 caused cellular senescence in endothelial cells following the induction of p16 by ROS accumulation and the activation of ERK (Figure 6). Our results might be correlated to oncogene- and stress-mediated activation of p16 transcription, which can be suppressed by members of the Ets family of transcription factors [26-27] and by Bmi-1, a polycomb transcription factor and member of the PRC1 complex [28]. Kim and Wong [29] also demonstrated that ROS-induced upregulation of p16 is linked to ERK1/2-dependent downregulation of Bmi-1. Therefore, although the mechanism for regulation of p16 transcription by GDF15 remains unclear, our findings suggested that GDF15 contributed to cellular senescence through the ROS-mediated p16 pathway in HAECs. Furthermore, GDF15-induced senescence in HAECs suggested that GDF15 released in senescent or irradiated endothelial cells might contribute to the pathogenesis of atherosclerosis *via* its pro-senescent activity.

## MATERIALS AND METHODS

### Cell lines and cell culture

HAECs were purchased from Life Technologies Corporation (Carlsbad, CA). Endothelial cell basal medium-2 (EBM-2) containing several growth factors and supplements, and Endothelial Cell Growth Medium (EGM-2) were purchased from Cambrex Bio Science, Inc. (Walkersville, MD). HAECs were cultured in EGM-2 media at 37°C in a 5% CO_2_ humidified incubator. When the subcultures reached 80-90% confluence, serial passaging was performed by trypsinization. For the experiments, cells were used in either passage 5-7 (PD < 24; young) or passage 12-14 (PD > 48; old).

### Materials

Recombinant human GDF15 (rhGDF15) was purchased from PeproTech (Rocky Hill, NJ). Antibodies against GDF15, p21, p16, and β-actin were purchased from Santa Cruz Biotechnology, Inc. (Santa Cruz, CA). Also, antibodies against phospho-Ataxia telangiectasia mutated (ATM) (serine 1981), phospho-p53 (serine 15), phospho-Rb (serine 807/811), phospho-ERK (threonine 202/tyrosine 204), and phospho-MEK (serine 217/221) were purchased from Cell Signaling Technology, Inc. (Danvers, MA). Silencing RNA (siRNA) against GDF15 was purchased from Dharmacon, Inc. (Chicago, IL).

### siRNA transfection

To knockdown GDF15, HAECs were transfected with GDF15 siRNAs (20 nM) using the TransIT-X2^®^ System transfection reagent (Mirus Bio LLC, Madison, WI) according to the manufacturer's protocol.

### Virus preparation and transduction

For overexpression of GDF15, the GDF15 transcript was cloned into a pLenti6/V5-TOPO vector (Life Technologies Corporation, Carlsbad, CA). The p16 and p53 shRNA retroviruses were prepared by transfection of pRetroSuper-p53sh and pRetroSuper-p16sh vectors into Human Embryonic Kidney 293 (HEK 293T) cells. After incubation for 3 days, the media were collected and centrifuged at 1,650 × *g* for 10 min. The viral solution was filtered using 0.45-μm filter membranes and concentrated with Vivaspin^®^ 20 centrifugal concentrators (Sartorius, Göttingen, Germany). Cells were then transduced with either the p16 shRNA or the p53 shRNA retrovirus. After incubation for 48 h, the p16 or p53 shRNA retrovirus-transduced cells were treated with 100 mg/ml recombinant GDF15 protein for 4 days.

### Reverse transcription-polymerase chain reaction (RT-PCR) and real-time PCR

Total RNA was extracted from HAECs using TRI-Reagent^®^ (Molecular Research Center, Cincinnati, OH) according to the manufacturer's protocols, and RNA concentrations were determined by measuring absorbance at 260 nm. A SensiFAST^TM^ cDNA synthesis kit from Bioline (BIO-65054, Taunton, MA) was used to synthesize cDNA. Real-time quantitative PCR analysis was performed using a CFX96 Touch™ Real-Time PCR Detection System (Bio-Rad, Hercules, CA). The PCR protocol included initial denaturation for 2 min at 95°C followed by 45 cycles of 95°C for 10 s, 60°C for 5 s, and 72°C for 12 s. Results were analyzed using CFX Manager™ software, version 2.1. Sequence-specific primers for GDF15, p53, p16 and GAPDH were from Bioneer Inc. (Daejeon, Korea).

### Protein extraction and western blot analysis

HAECs were seeded in 60-mm dishes and incubated for 24 h in EGM-2 media. Cells were washed with ice-cold phosphate-buffered saline (PBS), lysed in 50 μl of ice-cold RIPA buffer (25 mM Tris-HCl, pH 7.4, 150 mM NaCl, 1% NP-40, 0.5% sodium deoxycholate, 0.5% sodium dodecyl sulfate (SDS), 1 mM Na_3_VO_4_, 5 mM NaF, and 1 mM phenylmethylsulfonyl fluoride), and collected by scraping with a rubber policeman. For western blot analysis, proteins (30 μg) were separated on SDS-polyacrylamide gels and transferred to nitrocellulose membranes. One of the specific antibodies was applied to the membranes and the proteins were visualized using enhanced chemiluminescence. The membranes were then re-probed with β-actin antibody to serve as a protein loading control.

### Immunofluorescence

For immunofluorescence, HAECs were seeded in 12-well dishes on glass coverslips and incubated for 24 h in EGM-2 media. HAECs were fixed in 3.7% formaldehyde/1% fetal bovine serum (FBS)/PBS for 15 min at room temperature (RT), permeabilized with ice-cold 0.5% Triton X-100/PBS for 5 min, and blocked with 0.5% BSA/PBS for 1 h at RT. The cells were incubated overnight at 4°C with primary antibodies against GDF15. Cells were washed and incubated at RT with FITC-conjugated goat anti-rabbit IgG for 1 h and then stained for F-actin with Rhodamine-conjugated phalloidin (Life Technologies Corporation, Carlsbad, CA). Glass coverslips were attached to the glass slides with mounting solution (Vectashield; Vector Laboratories, Burlingame, CA) containing 6-diamidino-2-phenylindole (DAPI).

### Assessment of cellular ROS and mitochondrial biogenesis

Cellular ROS levels were detected using 2′,7′-Dichlorodihydrofluorescein diacetate (DCFDA; Cayman Chemical, Michigan). To analyze mitochondrial biogenesis, mitochondrial membrane potential (MMP) was assessed using JC-1 dye (Life Technologies Corporation, Carlsbad, CA). HAECs were trypsinized and mixed with 1% FBS/PBS including 10 μM DCFDA and JC-1 dye for 10 min. Stained cells were analyzed by the CyFlow^®^ Cube 6 flow cytometry system (Sysmex Partec, Norderstedt, Germany).

### SA-β-gal staining

Cellular SA-β-gal activity was measured as previously described [30]. Cells were grown to a density of 2 × 10^4^ cells in 35-mm culture dishes, washed with PBS, and fixed with 3.7% (v/v) paraformaldehyde for 10 min at RT. The cells were incubated with staining solution containing 1 mg/ml 5-bromo-4-chloro-3-indolyl-β-D-galactoside, 40 mM citric acid-sodium phosphate (pH 6.0), 5 mM potassium ferricyanide, 5 mM potassium ferrocyanide, 150 mM NaCl, and 2 mM MgCl_2_ for 16 h at 37°C. The SA-β-gal stained cells were washed with PBS, counter-stained with 1% eosin solution for 5 min, and then washed twice with ethanol. The percentage of blue cells per every 400 cells observed under a light microscope was calculated.
